# Association Between Medicare Expenditures and Adverse Events for Patients With Acute Myocardial Infarction, Heart Failure, or Pneumonia in the United States

**DOI:** 10.1001/jamanetworkopen.2020.2142

**Published:** 2020-04-07

**Authors:** Yun Wang, Noel Eldridge, Mark L. Metersky, Nancy Sonnenfeld, David Rodrick, Jonathan M. Fine, Sheila Eckenrode, Deron H. Galusha, Anila Tasimi, David R. Hunt, Susannah M. Bernheim, Sharon-Lise T. Normand, Harlan M. Krumholz

**Affiliations:** 1 Department of Biostatistics, Harvard T.H. Chan School of Public Health, Boston, Massachusetts; 2 Agency for Healthcare Research and Quality, Department of Health and Human Services, Washington, DC; 3 Pulmonary, Critical Care and Sleep Medicine, Department of Medicine, University of Connecticut School of Medicine, Farmington; 4 Centers for Medicare & Medicaid Services, Department of Health and Human Services, Washington, DC; 5 Asthma, Pulmonary and Critical Medicine, Norwalk Hospital, Norwalk, Connecticut; 6 Qualidigm, Wethersfield, Connecticut; 7 General Internal Medicine, Department of Internal Medicine, Yale School of Medicine, New Haven, Connecticut; 8 Office of the National Coordinator for Health Information Technology, Department of Health and Human Services, Washington, DC; 9 Department of Health Care Policy, Harvard Medical School, Boston, Massachusetts

## Abstract

**Importance:**

Studies have shown that adverse events are associated with increasing inpatient care expenditures, but contemporary data on the association between expenditures and adverse events beyond inpatient care are limited.

**Objective:**

To evaluate whether hospital-specific adverse event rates are associated with hospital-specific risk-standardized 30-day episode-of-care Medicare expenditures for fee-for-service patients discharged with acute myocardial infarction (AMI), heart failure (HF), or pneumonia.

**Design, Setting, and Participants:**

This cross-sectional study used the 2011 to 2016 hospital-specific risk-standardized 30-day episode-of-care expenditure data from the Centers for Medicare & Medicaid Services and medical record–abstracted in-hospital adverse event data from the Medicare Patient Safety Monitoring System. The setting was acute care hospitals treating at least 25 Medicare fee-for-service patients for AMI, HF, or pneumonia in the United States. Participants were Medicare fee-for-service patients 65 years or older hospitalized for AMI, HF, or pneumonia included in the Medicare Patient Safety Monitoring System in 2011 to 2016. The dates of analysis were July 16, 2017, to May 21, 2018.

**Main Outcomes and Measures:**

Hospitals’ risk-standardized 30-day episode-of-care expenditures and the rate of occurrence of adverse events for which patients were at risk.

**Results:**

The final study sample from 2194 unique hospitals included 44 807 patients (26.1% AMI, 35.6% HF, and 38.3% pneumonia) with a mean (SD) age of 79.4 (8.6) years, and 52.0% were women. The patients represented 84 766 exposures for AMI, 96 917 exposures for HF, and 109 641 exposures for pneumonia. Patient characteristics varied by condition but not by expenditure category. The mean (SD) risk-standardized expenditures were $22 985 ($1579) for AMI, $16 020 ($1416) for HF, and $16 355 ($1995) for pneumonia per hospitalization. The mean risk-standardized rates of occurrence of adverse events for which patients were at risk were 3.5% (95% CI, 3.4%-3.6%) for AMI, 2.5% (95% CI, 2.5%-2.5%) for HF, and 3.0% (95% CI, 2.9%-3.0%) for pneumonia. An increase by 1 percentage point in the rate of occurrence of adverse events was associated with an increase in risk-standardized expenditures of $103 (95% CI, $57-$150) for AMI, $100 (95% CI, $29-$172) for HF, and $152 (95% CI, $73-$232) for pneumonia per discharge.

**Conclusions and Relevance:**

Hospitals with high adverse event rates were more likely to have high 30-day episode-of-care Medicare expenditures for patients discharged with AMI, HF, or pneumonia.

## Key Points

**Question:**

What is the association between 30-day episode-of-care expenditures and in-hospital adverse events?

**Findings:**

This cross-sectional study of 44 807 patients, which linked the 2011 to 2016 hospital-specific risk-standardized 30-day episode-of-care expenditure data from the Centers for Medicare & Medicaid Services and medical record–abstracted in-hospital adverse event data from the Medicare Patient Safety Monitoring System, found that hospitals with high adverse event rates were more likely to have high 30-day episode-of-care Medicare expenditures for patients discharged with acute myocardial infarction, heart failure, or pneumonia.

**Meaning:**

This study suggests that hospitals with higher adverse event rates are more likely to have higher costs for acute myocardial infarction, heart failure, or pneumonia.

## Teaser

This cross-sectional study evaluates whether hospital-specific adverse event rates are associated with hospital-specific risk-standardized 30-day episode-of-care Medicare expenditures for fee-for-service patients discharged with acute myocardial infarction, heart failure, or pneumonia.

## Introduction

The US health care system is moving toward high-value care, with the goal of producing the best health outcomes at the lowest cost.^1,2^ Reducing both expenditures and hospital-acquired adverse events are 2 important aspects of this goal^3,4^ because health care expenditures are projected to increase faster than the US gross domestic product over the 2015 to 2025 period.^5^ Studies^6,7,8,9,10,11,12,13,14,15,16^ show that adverse events are associated with prolonged length of hospital stay, high mortality, unplanned readmissions, and deteriorating health status and quality of life of patients, all of which are associated with increased expenditures. However, few empirical studies have linked adverse events and expenditures across a large number of institutions.

A conceptual association between adverse events and expenditures could be that patients who have in-hospital adverse events may require additional expenditures to treat these adverse events. Such additional expenditures may also occur after discharge. Nevertheless, restricted by available data, previous studies were limited by the use of only a small number of measures^17,18^ and were largely focused on inpatient cost.^9,11,19,20,21,22,23,24,25,26,27^ Information is needed to examine the association between hospital performance on adverse events and hospital performance on episode-of-care expenditures within a standard period after admission in a contemporary and national cohort.

Accordingly, we sought to investigate the association at the hospital level between in-hospital adverse events and 30-day episode-of-care Medicare expenditures for Medicare fee-for-service patients with acute myocardial infarction (AMI), heart failure (HF), or pneumonia, 3 common conditions among older adults. The study used 2 unique national data sets, the hospital-specific Medicare 30-day episode-of-care expenditure data from the Centers for Medicare & Medicaid Services (CMS) and the adverse event data from the Medicare Patient Safety Monitoring System (MPSMS) to conduct this analysis. The 30-day Medicare episode-of-care expenditure data include all-source Medicare payments directly associated with care for individual services. It was the first database of its kind to be made available, and the MPSMS data represent the nation’s largest randomly selected hospital medical record–abstracted adverse event database. The setting was acute care hospitals treating at least 25 Medicare fee-for-service patients for AMI, HF, or pneumonia in the United States. Participants were Medicare fee-for-service patients 65 years or older hospitalized for AMI, HF, or pneumonia included in the MPSMS in 2011 to 2016. The dates of analysis were July 16, 2017, to May 21, 2018. In addition, we identified the best-performing hospitals in both expenditures and adverse events to represent high-value health care hospitals and assessed their characteristics.^1,28^

## Methods

### Study Sample

The institutional review board at Solutions IRB^29^ deemed that the requirement for informed consent could be waived for this cross-sectional study. The institutional review board at Solutions IRB reviewed the study protocol and granted a waiver of informed consent for the use of the deidentified database. This study followed the Strengthening the Reporting of Observational Studies in Epidemiology (STROBE) reporting guideline for observational studies.^30^

The Medicare expenditure data for AMI, HF, and pneumonia are available at the individual hospital level from the Hospital Compare website.^31^ The data include hospital-specific risk-standardized Medicare expenditures for episodes of care, starting with inpatient admission to a short-term acute care facility and extending 30 days after admission for Medicare fee-for-service patients (eAppendix 1 and eTable 1 in the Supplement). The geographic differences and policy adjustments in payment rates were removed. The CMS pooled expenditure data from a 3-year period to ensure that each hospital had sufficient discharges (ie, cases). Reporting periods used were from July 1, 2011, through June 30, 2014, from July 1, 2012, through June 30, 2015, and from July 1, 2013, through June 30, 2016. To include the maximum number of hospitals, we combined three 3-year-period data sets into a single multiple-year data set from July 1, 2011, to June 30, 2016. If a hospital was in multiple periods, we averaged its expenditure weighted by its average number of discharges in each period.

The MPSMS data, described elsewhere,^8,13,15,32,33,34,35,36,37,38^ are available at the individual patient level. The data include patient demographic, clinical, and comorbidity information and 21 in-hospital adverse event measures (eTable 2 in the Supplement) jointly developed by federal agencies and private health care organizations.^39,40^ Approximately 34 000 records were selected randomly from 1400 hospitals in 2011, 27 200 records from 1110 hospitals in 2012, 17 900 records from 730 hospitals in 2013, 25 300 records from 836 hospitals in 2014, 29 300 records from 1626 hospitals in 2015, and 29 800 records from 1190 hospitals in 2016. Hospitals were randomly selected and contributed approximately equal numbers of randomly selected medical records to the MPSMS. Medical record abstraction was conducted at the CMS Clinical Data Abstraction Center. Based on 80-monthly reabstractions, the agreement between abstraction and reabstraction ranged from 94% to 99% for data elements used to identify adverse events. To align the CMS and MPSMS data, we restricted the final cohort to Medicare patients discharged with AMI, HF, or pneumonia from a short-term acute care hospital in the United States from July 1, 2011, through June 30, 2016.

### Patient and Hospital Characteristics

Patient characteristics for the MPSMS data were obtained from medical records, and hospital characteristics were obtained from the American Hospital Association’s 2015 Annual Survey Database (eAppendix 2 in the Supplement). An Elixhauser Comorbidity Index score was calculated for each patient in the MPSMS sample. The score ranged from 0 to 29, with a score of 0 indicating no major comorbidities and a a score of 29 indicating the highest number of comorbidities. We then aggregated the score at the hospital level to represent hospital-specific patient complexity. An additional variable included was a fully electronic health record (yes or no) as assessed by the MPSMS data to reflect a hospital’s adoption of such a system.^35^

### Outcomes and In-Hospital Adverse Events

The primary outcome was hospital-specific risk-standardized 30-day episode-of-care Medicare expenditures, which combine Medicare payments directly associated with care for patients during their initial hospitalization and Medicare payments directly associated with continued care after discharge but within 30 days after admission from the initial hospitalization (eTable 1 in the Supplement). The CMS measures the initial hospitalization expenditures from the date of admission and post–acute care expenditures from the date of discharge for patients who were discharged alive. We used the hospital-specific risk-standardized rate of occurrence of adverse events as a proxy to measure the hospital performance on adverse events. Specifically, using the CMS risk-standardized method for profiling hospitals (eAppendix 3 in the Supplement), we fitted a mixed model with a Poisson link function to model the number of adverse events as a function of patients’ age, sex, and comorbidities. The number of exposures for which patients were at risk was the offset in the model. Using this model, a hospital-specific risk-standardized rate of occurrence of adverse events was estimated for each hospital. We then linked the risk-standardized adverse event measurement with the CMS hospital-specific risk-standardized expenditure data at the hospital level.

Our second outcome was high-value hospitals, defined as hospitals with both risk-standardized expenditures and the risk-standardized rate of occurrence of adverse events in the lowest quartile (<25th percentile). Because the classification of high-value care varies by condition, the range of this outcome is from 0 to 3, corresponding to none, 1, 2, and all 3 conditions in high-value care.

### Statistical Analysis

Each hospital was classified into 1 of the following 3 mutually exclusive categories based on its risk-standardized 30-day expenditures: (1) low if the expenditures were in the lowest quartile (<25th percentile), (2) high if the expenditures were in the highest quartile (>75th percentile), and (3) average if otherwise.^41,42^ We then performed a descriptive analysis to show patient and hospital characteristics and adverse event measurement across the 3 categories.

To evaluate the association between expenditures and adverse events at the hospital level, we fitted a linear regression model to link hospital-specific risk-standardized expenditures to the hospital-specific risk-standardized rate of occurrence of adverse events, with and without adjustment for hospital characteristics, including the hospital-specific Elixhauser Comorbidity Index score. The model was fitted for AMI, HF, and pneumonia separately.

To address potential uncertainty in the estimates of expenditures and adverse events, we conducted bootstrapping analyses. Because the expenditure data were only available at the hospital level, parametric bootstrapping was used to generate 2000 random data points based on the hospital-specific point and interval estimates in the expenditure data. The inverse of variance of these bootstrapped data points was used to weight by their precision in the regression analyses described above. Because the MPSMS data were at the individual patient level, we used nonparametric bootstrapping with replacement to generate 2000 random data sets using the method developed for the CMS outcome measurements.^43^ For each sub–data set and each hospital, we then calculated the risk-standardized rate of occurrence of adverse events described previously and fitted the above regressions to obtain a distribution and 95% CI for the estimate of the association between a hospital’s expenditures and the adverse event measure. To align with the CMS method for outcome measurements that restricts the analysis to hospitals with at least 25 discharges in the expenditure data, we conducted additional analyses by restricting the sample to hospitals with at least 25 adverse events for which patients were at risk over the study period.

Finally, we fitted a negative binomial regression model to assess hospital characteristics associated with high-value hospitals weighted by the hospital-specific number of exposures for which patients were at risk. Analyses were conducted using SAS, version 9.4, 64-bit (SAS Institute Inc).

## Results

### Study Sample

The final study sample based on linked CMS and MPSMS data across 2194 unique hospitals included 44 807 patients (26.1% with AMI, 35.6% with HF, and 38.3% with pneumonia), with a mean (SD) age of 79.4 (8.6) years, and 52.0% were women. The patients represented 84 766 exposures for AMI, 96 917 exposures for HF, and 109 641 exposures for pneumonia. Patient characteristics varied by condition but not by expenditure category. The mean (SD) ages were 78.2 (8.7) years for AMI, 80.2 (8.5) years for HF, and 79.1 (8.6) years for pneumonia, and women accounted for 47.0%, 54.7%, and 52.3% for each condition, respectively (Table 1). Hospitals that had high proportions of patients with coronary artery disease, kidney disease, and diabetes and hospitals that performed coronary artery bypass graft surgery had higher hospital-specific risk-standardized expenditures for all 3 conditions (Table 1).

**Table 1.** Patient and Hospital Characteristics and Patient Outcomes by Condition and Expenditure Category^a^

Variable	AMI and expenditure category^b^				HF and expenditure category^c^				Pneumonia and expenditure category^d^			
	Overall	Low	Average	High	Overall	Low	Average	High	Overall	Low	Average	High
**Patient characteristics**^**e**^												
No. of patients	11 715	2085	5754	3876	15 947	5482	7191	3274	17 145	2257	3808	11 080
Age, mean (SD), y	78.2 (8.7)	78.7 (8.9)	78.1 (8.7)	78.2 (8.7)	80.2 (8.5)	80.1 (8.5)	80.2 (8.5)	80.2 (8.5)	79.1 (8.6)	79.3 (8.5)	79.3 (8.7)	79.1 (8.5)
Female	47.0	48.7	46.8	46.5	54.7	56.1	54.4	53.0	52.3	53.2	51.7	52.3
White	85.8	88.3	85.8	84.4	84.1	84.5	85.1	81.1	86.2	86.8	88.7	85.2
Black	8.5	7.3	9.0	8.5	10.9	11.2	10.5	11.0	8.0	6.3	7.3	8.6
Other race	5.7	4.4	5.3	7.2	5.1	4.3	4.3	8.0	5.9	7.0	4.1	6.3
History of HF	48.1	46.4	48.1	49.1	98.5	98.7	98.4	98.5	40.9	40.6	39.3	41.5
Obesity	23.2	22.4	23.3	23.5	28.4	27.2	29.1	28.8	19.3	16.6	17.9	20.3
Coronary artery disease	97.4	97.2	97.5	97.4	65.6	62.0	66.3	70.1	44.0	40.2	43.2	45.0
Kidney disease	38.6	38.4	38.3	39.1	52.6	49.9	53.9	54.4	37.0	29.4	34.0	40.0
Cerebrovascular disease	23.4	25.3	22.9	23.1	23.1	21.9	24.1	22.8	22.0	18.2	21.4	22.9
COPD	26.6	27.3	26.0	27.4	43.3	42.3	44.3	42.6	51.1	52.6	51.0	51.0
All cancer	19.9	19.9	19.8	19.9	21.4	20.3	22.0	21.9	27.8	22.5	26.4	29.4
Diabetes	42.6	40.6	42.6	43.7	48.5	48.2	48.8	48.3	37.2	37.0	36.0	37.6
Smoking	18.8	17.7	19.4	18.7	15.2	15.9	15.3	13.8	19.0	20.5	19.8	18.4
**Patient outcomes**^**e**^												
Length of stay, median (IQR), d	3 (2-5)	3 (2-5)	3 (2-5)	3 (2-6)	4 (2-6)	3 (2-5)	4 (2-6)	4 (3-6)	4 (3-7)	4 (3-6)	4 (3-6)	5 (3-7)
In-hospital mortality	7.3	8.4	7.0	7.2	3.8	4.1	3.8	3.3	8.7	6.2	7.8	9.6
Adverse events												
No. of exposures during a hospitalization, mean (SD)	7.2 (2.5)	6.9 (2.2)	7.2 (2.4)	7.4 (2.6)	6.1 (1.4)	6.0 (1.3)	6.1 (1.5)	6.2 (1.5)	6.4 (1.3)	6.1 (1.0)	6.3 (1.2)	6.5 (1.3)
Adverse event rate	3.3	2.8	3.2	3.7	2.4	2.1	2.5	2.9	2.9	2.0	2.5	3.2
**Hospital characteristics**												
No. of hospitals	1647	291	834	522	2029	640	952	437	2060	223	444	1393
Major teaching	9.7	7.6	10.7	9.4	7.7	3.1	9.8	9.8	7.5	0.9	2.9	10.1
Accredited by The Joint Commission	84.6	82.1	85.6	84.5	79.5	70.6	82.7	85.6	78.8	49.8	76.4	84.2
Private and not for profit	67.2	66.7	69.3	64.0	63.7	59.2	66.0	65.2	63.0	49.8	57.4	66.8
Rural setting	27.9	37.1	30.5	18.6	27.9	37.5	28.2	13.3	28.0	31.4	35.6	25.1
Perform CABG surgery	46.4	45.0	47.2	45.8	36.9	19.5	42.0	51.3	35.9	4.0	23.2	45.0
Perform cardiac catheterization or PCI	64.2	63.6	64.4	64.2	51.9	33.0	58.8	64.5	50.5	10.3	37.4	61.1
Fully electronic health record	9.9	7.2	11.2	9.4	9.1	8.6	10.3	7.1	8.9	5.8	8.3	9.6
Adult cardiology services	78.9	79.0	79.3	78.2	69.0	55.8	74.9	75.3	67.5	28.7	61.3	75.7
With case management	87.0	89.4	87.1	85.6	83.7	82.7	84.8	82.8	83.5	70.4	83.6	85.5
Community outreach	77.6	77.3	78.5	76.3	72.6	67.7	75.7	72.8	71.8	55.6	67.6	75.8
Perform MRI	82.5	82.5	83.3	81.2	77.6	74.7	79.4	77.8	77.7	59.2	77.7	80.6
Safety-net hospital	20.8	25.1	20.5	18.8	23.8	32.7	21.9	15.1	25.0	37.7	28.8	21.7
Beds, median, No. (IQR)	204 (122-341)	203 (116-357)	208 (118-344)	203 (135-335)	167 (90-290)	111 (58-203)	181 (101-308)	238 (145-365)	165 (87-288)	68 (42-126)	115 (60-203)	203 (116-345)
Adjusted all-cause length of stay, median (IQR), d	4.6 (4.1-5.3)	4.6 (4.0-5.4)	4.5 (4.0-5.3)	4.6 (4.1-5.2)	4.5 (3.9-5.3)	4.4 (3.6-5.5)	4.5 (3.9-5.2)	4.7 (4.2-5.3)	4.5 (3.9-5.3)	4.3 (3.5-6.0)	4.4 (3.7-5.3)	4.6 (4.0-5.3)

Abbreviations: AMI, acute myocardial infarction; CABG, coronary artery bypass graft; COPD, chronic obstructive pulmonary disease; HF, heart failure; IQR, interquartile range; MRI, magnetic resonance imaging; PCI, percutaneous coronary intervention.

^a^ Unless otherwise specified, percentages are given for the variables.

^b^ Expenditure category ranges: overall, $17 971-$31 134; low, $17 971-$21 882; average, $21 883-$23 983; high, $23 984-$31 134.

^c^ Expenditure category ranges: overall, $12 599-$25 127; low, $12 599-$15 056; average, $15 057-$16 843; high, $16 844-$25 127.

^d^ Expenditure category ranges: overall, $11 566-$37 193; low, $11 566-$15 087; average, $15 088-$17 328; high, $17 329-$37 193.

^e^ Based on data abstracted from the Medicare Patient Safety Monitoring System.

### Expenditures and Adverse Events

The mean (SD) risk-standardized expenditures were $22 985 ($1579) for AMI, $16 020 ($1416) for HF, and $16 355 ($1995) for pneumonia per hospitalization (eFigure 1 in the Supplement). Hospitals with high expenditures for 1 condition were also likely to have high expenditures for other conditions (eFigure 2 in the Supplement). The hospital-specific median numbers of adverse events were 40 (interquartile range [IQR], 19-66) for AMI, 39 (IQR, 19-65) for HF, and 47 (IQR, 20-75) for pneumonia. Each patient had a mean of 7.1 (range, 3-19) exposures for AMI, 6.1 (range, 3-17) exposures for HF, and 6.4 (range, 3-17) exposures for pneumonia.

**Figure 1.**
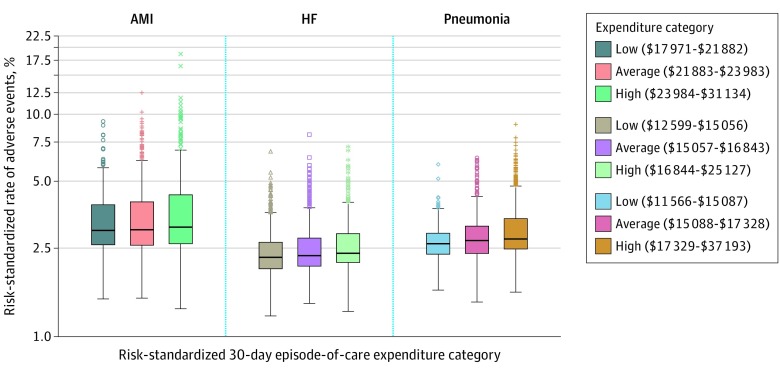
Box and Whisker Plots of the Distribution of Hospital-Specific Risk-Standardized Adverse Events by Condition and Hospital-Specific Risk-Standardized Medicare 30-Day Episode-of-Care Expenditure Category The height of the box represents the interquartile range (IQR), the horizontal line in the box interior represents the median, the whiskers represent the 1.5 IQR of the 25th quartile or the 1.5 IQR of the 75th quartile, and the circles, plus signs, Xs, triangles, squares, and diamonds represent outliers. Each point represents an individual hospital. AMI indicates acute myocardial infarction; HF, heart failure.

The mean risk-standardized rates of occurrence of adverse events for which patients were at risk were 3.5% (95% CI, 3.4%-3.6%) for AMI, 2.5% (95% CI, 2.5%-2.5%) for HF, and 3.0% (95% CI, 2.9%-3.0%) for pneumonia and varied by expenditure group (Figure 1). Hospitals with a high number of adverse events in 1 condition were likely to have a high number of adverse events in other conditions except for AMI vs pneumonia (eFigure 3 in the Supplement).

**Figure 2.**
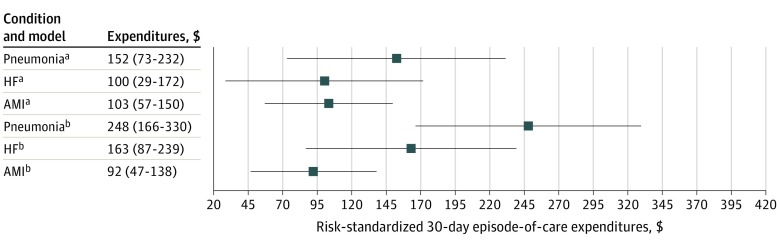
Change in Hospital-Specific Risk-Standardized Medicare 30-Day Episode-of-Care Expenditures per Discharge for 1–Percentage Point Increase in the Adverse Event Rate by Condition AMI indicates acute myocardial infarction; HF, heart failure; and the numbers in parentheses represent the range. ^a^Adjusted for hospital characteristics. ^b^Unadjusted for hospital characteristics.

The risk-standardized rate of occurrence of adverse events was associated with the risk-standardized expenditures for all 3 conditions (eFigure 4 in the Supplement), with or without adjustment for hospital characteristics (Figure 2). An increase by 1 percentage point in the rate of occurrence of adverse events was associated with an increase in risk-standardized expenditures of $103 (95% CI, $57-$150) for AMI, $100 (95% CI, $29-$172) for HF, and $152 (95% CI, $73-$232) for pneumonia per discharge for the specified condition (Figure 2 and eTable 3 in the Supplement).

The additional analyses, which restricted the sample to hospitals with at least 25 adverse events, showed an even stronger association for AMI and HF. An increase by 1 percentage point in the rate of occurrence of adverse events was associated with an increase in risk-standardized expenditures of $114 (95% CI, $63-$166) for AMI and $116 (95% CI, $39-$193) for HF per discharge. This association was reduced for pneumonia ($132; 95% CI, $49-$216) (eFigure 5 in the Supplement).

### High-Value Hospitals

The numbers of hospitals classified as providing high-value care were 73 of 1647 (4.4%) for AMI, 189 of 2029 (9.3%) for HF, and 71 of 2060 (3.4%) for pneumonia, and they treated 5.6% of patients with AMI, 6.3% of patients with HF, and 5.9% of patients with pneumonia. Together, they represented 291 of 2194 unique hospitals (13.3%), of which 2 (0.7%) delivered high-value care for all 3 conditions, 38 (13.1%) for 2 conditions, and 251 (86.3%) for 1 condition. High-value hospital characteristics varied by condition (Table 2). Hospitals with case management, safety-net hospitals, and hospitals with a fully electronic health record were more likely to be classified as delivering high-value care (eFigure 6 in the Supplement).

**Table 2.** Hospital Characteristics Associated With High-Value Care by Condition

Variable	Hospitals classified as high value, No. (%)					
	AMI (n = 1647)		HF (n = 2029)		Pneumonia (n = 2060)	
	None^a^	≥1^b^	None^a^	≥1^b^	None^a^	≥1^b^
No. of hospitals	1574 (95.6)	73 (4.4)	1840 (90.7)	189 (9.3)	1989 (96.6)	71 (3.4)
Major teaching	156 (9.9)	4 (5.5)	151 (8.2)	5 (2.6)	154 (7.7)	1 (1.4)
Accredited by The Joint Commission	1337 (84.9)	57 (78.1)	1482 (80.5)	131 (69.3)	1591 (80.0)	32 (45.1)
Private and not for profit	1060 (67.3)	46 (63.0)	1180 (64.1)	112 (59.3)	1264 (63.5)	33 (46.5)
Rural setting	434 (27.6)	25 (34.2)	503 (27.3)	63 (33.3)	555 (27.9)	22 (31.0)
Perform CABG surgery	744 (47.3)	20 (27.4)	719 (39.1)	30 (15.9)	736 (37.0)	3 (4.2)
Perform cardiac catheterization or PCI	1018 (64.7)	39 (53.4)	992 (53.9)	61 (32.3)	1032 (51.9)	8 (11.3)
Fully electronic health record	158 (10.0)	5 (6.8)	162 (8.8)	22 (11.6)	180 (9.0)	4 (5.6)
Adult cardiology services	1247 (79.2)	52 (71.2)	1299 (70.6)	100 (52.9)	1371 (68.9)	19 (26.8)
With case management	1372 (87.2)	61 (83.6)	1547 (84.1)	151 (79.9)	1662 (83.6)	57 (80.3)
Community outreach	1227 (78.0)	51 (69.9)	1349 (73.3)	123 (65.1)	1443 (72.5)	37 (52.1)
Perform MRI	1305 (82.9)	54 (74.0)	1439 (78.2)	135 (71.4)	1553 (78.1)	47 (66.2)
Safety-net hospital	320 (20.3)	22 (30.1)	421 (22.9)	62 (32.8)	480 (24.1)	34 (47.9)
Beds >100, No.	1311 (83.3)	54 (74.0)	1367 (74.3)	82 (43.4)	1426 (71.7)	18 (25.4)
Adjusted all-cause length of stay >5 d	524 (33.3)	28 (38.4)	606 (32.9)	54 (28.6)	649 (32.6)	19 (26.8)

Abbreviations: AMI, acute myocardial infarction; CABG, coronary artery bypass graft; HF, heart failure; MRI, magnetic resonance imaging; PCI, percutaneous coronary intervention.

^a^ Did not meet high-value care criteria.

^b^ Met high-value care criteria.

## Discussion

This study used the hospital-specific risk-standardized rate of occurrence of adverse events as a proxy measurement of the hospital performance on adverse events. We found that the hospital performance on adverse events was associated with hospital-specific risk-standardized 30-day episode-of-care expenditures for patients with AMI, HF, or pneumonia. This finding suggests that investment in reducing adverse events may provide substantial savings in Medicare cost. Although the rationale to reduce adverse events goes far beyond economics, we believe that empirical data from across the country demonstrated the alignment between adverse events and cost.

There are several possible explanations for our findings. Patients who developed in-hospital adverse events probably required more care or were at increased risk of mortality^7^ and were more likely to be readmitted,^15^ at least for AMI. The Office of Inspector General found that two-thirds of Medicare hospital costs associated with adverse events were the result of additional hospital stays necessitated within the same calendar month as the index hospitalization because of harm from the adverse event.^16^ Complications resulting from in-hospital adverse events may also cause additional adverse events after discharge, rendering these patients more likely to receive post–acute care services in skilled nursing facilities, home health care, and outpatient visits, as well as unplanned readmissions; consequently, such patients have higher 30-day episode-of-care risk-standardized expenditures compared with patients who do not develop an adverse event during their hospitalization.^16,44,45^ It is also possible that these patients were provided post–acute care services with higher rates of ambulatory care and follow-up with a condition-specific specialist after discharge, which are associated with additional expenditures.^46^

The present study based on medical record–abstracted adverse event information was a large population-based investigation to assess the association between hospital performance on adverse events and 30-day expenditures for an episode of care for AMI, HF, or pneumonia in a contemporary cohort of Medicare beneficiaries in the United States. The use of risk-standardized 30-day payment data allowed us to capture costs not only during an index hospitalization but also immediately after discharge, a period in which substantial variation in Medicare expenditures exists predominantly because of differential use of post–acute care services.^26^ Previous studies^16,23,47,48,49,50,51^ were restricted to in-hospital cost, but this study extends the cost from in-hospital to a 30-day standard period. For example, Zhan et al^48^ found that Medicare paid an extra $300 million in 2002 for 5 types of adverse events (pressure ulcer, iatrogenic pneumothorax, postoperative hematoma or hemorrhage, postoperative pulmonary embolism or deep vein thrombosis, and postoperative sepsis). Spector et al^49^ found that the occurrence of a hospital-acquired pressure ulcer was associated with an estimated $792 million in additional hospital costs that were incurred nationwide. Tsai et al^50^ found that patients who had major surgery at high-quality hospitals cost Medicare less than patients who had major surgery at low-quality institutions. Shamliyan and Kane^51^ found that hospitalizations associated with drug poisoning comprised 0.8% of all Medicare hospitalizations, with an annual hospital cost of $4 billion in 2008; in-hospital adverse drug events occurred during 5.3% of all Medicare hospitalizations. However, none of these studies captured expenditures for both inpatient and post–acute care services for AMI, HF, or pneumonia.

Reductions in adverse events often require investment in additional resources, which could increase a hospital’s overall budget and operating costs in the short term. However, from a long-term perspective, such an investment may reduce both Medicare expenditures and hospital costs, in addition to the primary objective of delivering safer care. The Office of Inspector General found that 84% of adverse events did not add to the Medicare payment for an inpatient stay.^16^ The reason is because these claims did not include diagnosis or procedure codes associated with the adverse events. Even if the claims included codes associated with the events, the codes often had no association with payments because the claims included other costly diagnoses or procedure codes that elevated the reimbursement to equivalent or higher amounts. Nevertheless, hospitals often must absorb the cost for these events. Researchers in Canada found savings of $9.1 million after implementing an infection prevention and control system that cost $6.7 million.^52^ Pettker et al^53^ reviewed liability claims at a single tertiary care teaching hospital for two 5-year periods (1998-2002 and 2003-2007) before and after implementing a safety program. They found that both liability claims (30 vs 14) and expenditures ($50.7 million vs $2.9 million) declined with the program.

### Limitations

This study has limitations. We focused on adverse events that occurred during the index hospitalization and not after discharge; therefore, some events may have been missed. However, Forster et al^54,55^ showed that adverse events frequently occur during the index hospitalization and adverse events that occur after hospital discharge are typically drug related. Restricted by the MPSMS data, we were unable to assess whether some of the measured adverse events have stronger associations with Medicare expenditures than others. It is possible that a proportion of the adverse events detected in the MPSMS may not be preventable, although each of the 21 in-hospital adverse event measures is characterized as being frequently preventable with the delivery of high-quality care. The study may also have underestimated the association between expenditures and adverse events because it is possible that some of the 21 adverse events may require care beyond a 30-day period. Limited by available expenditure data, we were unable to assess the expenditures from direct treatment of adverse events, and it is plausible that some expenditures may be associated with unmeasured confounding factors and that these expenditures may not be attributable to differences in adverse events. In addition, poor hospital performance on adverse events could be a marker of other systemic contributors and mechanisms, such as lower staffing ratios associated with care inefficiency and longer length of stay. Although the scope of this study constrained our ability to address these limitations in depth, future studies are warranted to elucidate them. Nevertheless, this study distinguishes itself by the breadth and standardization of events measured and its national scope.

## Conclusions

This study suggests that hospitals with poor performance on adverse events are likely to have high 30-day expenditures for AMI, HF, and pneumonia. These findings strengthen the evidence that adverse events may reflect the quality of hospital care and their reduction may be used as a mechanism for decreasing Medicare expenditures.

## Notes

Supplement. **eAppendix 1.** CMS Risk-Standardized Payment Method for Profiling Hospitals **eAppendix 2.** Patient and Hospital Characteristics **eAppendix 3.** CMS Risk-Standardized Outcome Method for Profiling Hospitals **eTable 1.** Sources of Expenditures Included in the CMS 30-Day All-Cause Risk-Standardized Data **eTable 2.** List of the 21 Adverse Event Measures in the Medicare Patient Safety Monitoring System **eTable 3.** Estimates From the Regression Analysis **eFigure 1.** Distributions of Medicare 30-Day Episode-of-Care Expenditures by AMI, HF, and Pneumonia **eFigure 2.** Relationship of Risk-Standardized Medicare 30-Day Episode-of-Care Expenditures by AMI, HF, and Pneumonia **eFigure 3.** Relationship of Risk-Standardized Rate of Adverse Events Among AMI, HF, and Pneumonia **eFigure 4.** Relationship Between Hospital-Specific Risk-Standardized Medicare 30-Day Episode-of-Care Expenditures and Adverse Events by Condition **eFigure 5.** Association Between Hospital-Specific Performance on Patient Safety and Hospital-Specific Performance on 30-Day Episode-of-Care Expenditures by AMI, HF, and Pneumonia (Hospitals With at Least 25 Adverse Events for Which Patients Were at Risk for Each Condition) **eFigure 6.** Hospital Characteristics Associated With High-Value Care
