# Supplementary material for: Association Between Medicare Expenditures and Adverse Events for Patients With Acute Myocardial Infarction, Heart Failure, or Pneumonia in the United States
Source: JAMA Netw Open. 2020 Apr 7;3(4):e202142. doi: 10.1001/jamanetworkopen.2020.2142 (PMC7139276; doi:10.1001/jamanetworkopen.2020.2142)
Supplement: Supplement. — eAppendix 1. CMS Risk-Standardized Payment Method for Profiling Hospitals eAppendix 2. Patient and Hospital Characteristics eAppendix 3. CMS Risk-Standardized Outcome Method for Profiling Hospitals eTable 1. Sources of Expenditures Included in the CMS 30-Day All-Cause Risk-Standardized Data eTable 2. List of the 21 Adverse Event Measures in the Medicare Patient Safety Monitoring System eTable 3. Estimates From the Regression Analysis eFigure 1. Distributions of Medicare 30-Day Episode-of-Care Expenditures by AMI, HF, and Pneumonia eFigure 2. Relationship of Risk-Standardized Medicare 30-Day Episode-of-Care Expenditures by AMI, HF, and Pneumonia eFigure 3. Relationship of Risk-Standardized Rate of Adverse Events Among AMI, HF, and Pneumonia eFigure 4. Relationship Between Hospital-Specific Risk-Standardized Medicare 30-Day Episode-of-Care Expenditures and Adverse Events by Condition eFigure 5. Association Between Hospital-Specific Performance on Patient Safety and Hospital-Specific Performance on 30-Day Episode-of-Care Expenditures by AMI, HF, and Pneumonia (Hospitals With at Least 25 Adverse Events for Which Patients Were at Risk for Each Condition) eFigure 6. Hospital Characteristics Associated With High-Value Care [file jamanetwopen-3-e202142-s001.pdf]

Supplementary Online Content

Wang Y, Eldridge N, Metersky ML, et al. Association between Medicare expenditures and adverse events for patients with acute myocardial infarction, heart failure, or pneumonia in the United States. *JAMA Netw Open*. 2020;3(4):e202142. doi:10.1001/jamanetworkopen.2020.2142

eAppendix 1. CMS Risk-Standardized Payment Method for Profiling Hospitals

eAppendix 2. Patient and Hospital Characteristics

eAppendix 3. CMS Risk-Standardized Outcome Method for Profiling Hospitals

eTable 1. Sources of Expenditures Included in the CMS 30-Day All-Cause Risk-Standardized Data

eTable 2. List of the 21 Adverse Event Measures in the Medicare Patient Safety Monitoring System

eTable 3. Estimates From the Regression Analysis

eFigure 1. Distributions of Medicare 30-Day Episode-of-Care Expenditures by AMI, HF, and Pneumonia

eFigure 2. Relationship of Risk-Standardized Medicare 30-Day Episode-of-Care Expenditures by AMI, HF, and Pneumonia

eFigure 3. Relationship of Risk-Standardized Rate of Adverse Events Among AMI, HF, and Pneumonia

eFigure 4. Relationship Between Hospital-Specific Risk-Standardized Medicare 30-Day Episode-of-Care Expenditures and Adverse Events by Condition

eFigure 5. Association Between Hospital-Specific Performance on Patient Safety and Hospital-Specific Performance on 30-Day Episode-of-Care Expenditures by AMI, HF, and Pneumonia (Hospitals With at Least 25 Adverse Events for Which Patients Were at Risk for Each Condition)

eFigure 6. Hospital Characteristics Associated With High-Value Care

This supplementary material has been provided by the authors to give readers additional information about their work.

© 2020 Wang Y et al. *JAMA Network Open*.

Supplementary Appendix

eAppendix 1.

CMS risk-standardized payment method for profiling hospitals: For a given condition, the 30-day risk-standardized payment is calculated as the ratio of *predicted* condition-specific payment to *expected* condition-specific payment, multiplied by the national unadjusted mean payment for that condition. The *predicted* condition-specific payment for each hospital was estimated given the same patient mix but an estimated hospital-specific intercept. The *predicted* condition-specific payment for each hospital is calculated by summing the predicted condition-specific payments for all patients in the hospital. The *expected* condition-specific payment for each hospital is estimated using its patient mix and the average of the hospital specific intercepts. The *expected* condition-specific payment for each hospital is obtained by summing the *expected* condition-specific payments for all patients in the hospital.

eAppendix 2.

Patient and Hospital Characteristics: Patient characteristics for the MPSMS data were obtained from medical records and included demographics (age, sex, and race), comorbidities (heart failure, obesity, coronary artery disease, renal disease, cerebrovascular disease, chronic obstructive pulmonary disease, cancer, diabetes), and smoking status. Hospital characteristics were obtained from the American Hospital Association's 2015 Annual Survey Database, including teaching status (teaching versus non-teaching), Joint Commission certification status (yes/no), geographic location (urban versus non-urban), ownership (private not-for profit versus others), bed size (continuous), performance of coronary artery bypass graft surgery (yes/no), percutaneous coronary intervention (yes/no), adult cardiology services (yes/no), case management (yes/no), community outreach (yes/no), safety-net hospital (yes/no), capable for magnetic resonance imaging scan (yes/no), and adjusted all-condition patient length of stays (days).

eAppendix 3.

© 2020 Wang Y et al. *JAMA Network Open*.

<u>CMS risk-standardized outcome method for profiling hospitals (1-4):</u> Using a hierarchical generalized linear modeling approach that accounts for patient characteristics and permits hospital-level random intercepts, CMS calculates a risk-standardized ratio, defined as hospital-specific "predicted" deaths divided by hospital-specific "expected" deaths, for each hospital. The "predicted" deaths in a hospital were estimated by using the same patient mix with a hospital-specific intercept and the "expected" deaths in a hospital were estimated by using the same patient mix but with an overall intercept. This ratio, multiplied by the overall national mortality rate, is the risk-standardized mortality rate for an index hospital. A higher than expected death rate is indicated when the risk-standardized rate is greater than the national rate. The same approach is used to calculate a risk-standardized rate for hospital readmissions.

Reference

1. Krumholz HM, Wang Y, Mattera JA, Wang Y-F, Han LF, Ingber MJ, Roman S, Normand S-LT. (2006). An administrative claims model suitable for profiling hospital performance based upon 30-day mortality rates among patients with an acute myocardial infarction. *Circulation* 113:1683-92.
2. Krumholz HM, Wang Y, Mattera JA, Wang Y-F, Han LF, Ingber MJ, Roman S, Normand S-LT. (2006). An administrative claims model suitable for profiling hospital performance based upon 30-day mortality rates among patients with heart failure. *Circulation* 113;1693-1701.
3. Bratzler W, Normand SLT, Wang Y, O'Donnell WJ, Metersky M, Han LF, Rapp MT, Krumholz HM (2011) An Administrative claims model for profiling hospital 30-day mortality rates for pneumonia patients, *PLoS ONE* 6(4):1-7.
4. Normand SLT, Wang Y, Krumholz HM. (2007). Assessing surrogacy of data sources for institutional comparisons. *Health Serv Outcomes Res Method* 7:79-96.

© 2020 Wang Y et al. *JAMA Network Open*.

e**Table 1**. Sources of expenditures included in the CMS 30-day all-cause risk-standardized data

Ambulatory Procedure/Imaging Center
Ancillary Services
Behavioral Health Care
Emergency Department
Emergency Medical Services
Home Care
Hospices
Hospital Inpatient
Hospital Outpatient
Long-term Care Facilities - Other
Rehabilitation Centers
Rural Health Care
Skilled Nursing Facilities/Nursing Homes
Other

© 2020 Wang Y et al. *JAMA Network Open*.

e**Table 2**. List of the 21 adverse event measures in the Medicare Patient Safety Monitoring System

**Adverse Event Measures for which Patients Were at Risk during Hospitalizations**
Adverse Events Associated with Digoxin
Adverse Events Associated with Hypoglycemic Agents
Adverse Events Associated with Heparin
Adverse Events Associated with Low Molecular Weight Heparin and Factor Xa Inhibitors
Adverse Events Associated with Warfarin
Hospital-Acquired Pressure Ulcers
Inpatient Falls
Central Line-Associated Blood Stream Infections
Postoperative Pneumonia
Hospital-Acquired Antibiotic-Associated Clostridium difficile
Catheter-Associated Urinary Tract Infections
Hospital-Acquired Methicillin-Resistant Staphylococcus aureus
Hospital-Acquired Vancomycin-Resistant Enterococcus
Ventilator-Associated Pneumonia
Adverse Events Associated with Hip Joint Replacement
Adverse Events Associated with Knee Joint Replacement
Mechanical Complications Associated with Central Lines
Postoperative Venous Thromboembolic Events
Postoperative Cardiac Events (Cardiac and Non-cardiac Surgeries)
Adverse Events Associated with Femoral Artery Puncture for Catheter Angiographic Procedures
Contrast Nephropathy Associated with Catheter Angiography

© 2020 Wang Y et al. *JAMA Network Open*.

eTable 3. Estimates from the regression analysis

Condition	Variable	Point Estimate	Standard Error	P Value
AMI	Intercept	22849.9	233.4	0.0000
AMI	Risk-standardized occurrence rate of adverse events (%)	103.4	23.6	0.0000
AMI	Major teaching hospital (y/n)	141.5	127.8	0.2682
AMI	Private and not for profit hospital (y/n)	-185.3	96.9	0.0561
AMI	Beds (#)	0.3	0.2	0.0713
AMI	Rural setting (y/n)	-499.8	82.3	0.0000
AMI	Accredited by Joint Commission (y/n)	70.5	105.5	0.5043
AMI	Perform CATH or PCI (y/n)	-208.2	150.2	0.1658
AMI	Perform coronary artery bypass graft surgery	-263.1	111.7	0.0186
AMI	Fully electronic health record (y/n)	137.8	123.2	0.2637
AMI	Adult cardiology services (y/n)	103.8	192.8	0.5904
AMI	With case management (y/n)	-255.7	271.6	0.3467
AMI	Community outreach (y/n)	11.1	140.3	0.9369
AMI	Perform MRI (y/n)	289.3	171.7	0.0923
AMI	Safety-net hospital (y/n)	-337.0	108.1	0.0019
AMI	Adjusted all-cause length of stay (day)	-68.4	18.0	0.0001
AMI	Average of Elixhauser score	69.2	52.4	0.1866
HF	Intercept	15683.7	175.8	0.0000
HF	Risk-standardized occurrence rate of adverse events (%)	100.1	36.5	0.0062
HF	Major teaching hospital (y/n)	-120.3	96.1	0.2109
HF	Private and not for profit hospital (y/n)	-170.2	69.9	0.0151
HF	Beds (#)	0.5	0.1	0.0000
HF	Rural setting (y/n)	-594.9	60.9	0.0000
HF	Accredited by Joint Commission (y/n)	256.4	72.8	0.0004
HF	Perform CATH or PCI (y/n)	314.4	95.9	0.0011
HF	Perform coronary artery bypass graft surgery	221.2	80.3	0.0059
HF	Fully electronic health record (y/n)	30.5	90.7	0.7369
HF	Adult cardiology services (y/n)	230.7	111.1	0.0379
HF	With case management (y/n)	-590.0	162.0	0.0003
HF	Community outreach (y/n)	92.7	96.6	0.3371
HF	Perform MRI (y/n)	46.0	114.6	0.6879
HF	Safety-net hospital (y/n)	-421.3	75.4	0.0000
HF	Adjusted all-cause length of stay (day)	-32.5	8.9	0.0003
HF	Average of Elixhauser score	74.7	3.9	0.0277
PN	Intercept	15801.7	219.8	0.0000
PN	Risk-standardized occurrence rate of adverse events (%)	152.4	40.4	0.0002
PN	Major teaching hospital (y/n)	-63.5	135.7	0.6398
PN	Private and not for profit hospital (y/n)	-103.0	90.6	0.2561
PN	Beds (#)	0.4	0.2	0.0068
PN	Rural setting (y/n)	-396.7	79.3	0.0000
PN	Accredited by Joint Commission (y/n)	407.0	93.0	0.0000
PN	Perform CATH or PCI (y/n)	395.1	124.7	0.0016
PN	Perform coronary artery bypass graft surgery	166.8	106.3	0.1168
PN	Fully electronic health record (y/n)	108.2	122.2	0.3761
PN	Adult cardiology services (y/n)	564.3	134.9	0.0000

© 2020 Wang Y et al. *JAMA Network Open*.

PN	With case management (y/n)	-639.8	200.4	0.0014
PN	Community outreach (y/n)	81.5	119.4	0.4951
PN	Perform MRI (y/n)	-053.5	149.0	0.7196
PN	Safety-net hospital (y/n)	-192.6	098.5	0.0507
PN	Adjusted all-cause length of stay (day)	-048.6	011.0	0.0000
PN	Average of Elixhauser score	-086.2	046.9	0.0661

AMI=Acute Myocardial Infarction, HF=Heart Failure, PN=Pneumonia

© 2020 Wang Y et al. *JAMA Network Open*.

eFigure 1. Distributions of Medicare 30-day episode-of-care expenditures by AMI, HF, and pneumonia

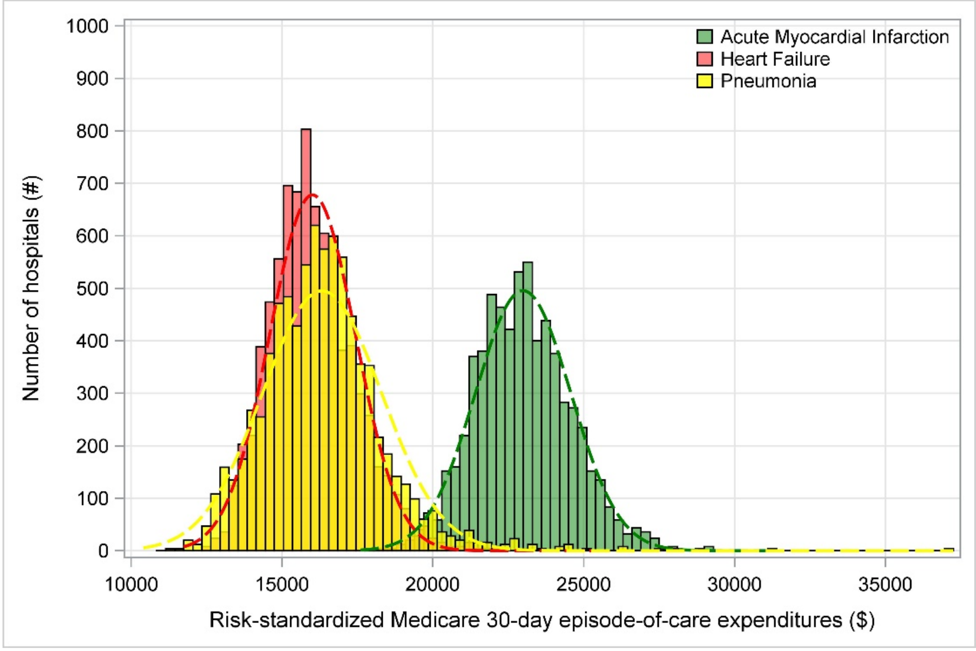

© 2020 Wang Y et al. *JAMA Network Open*.

eFigure 2. Relationship of risk-standardized Medicare 30-day episode-of-care expenditures by AMI, HF, and pneumonia

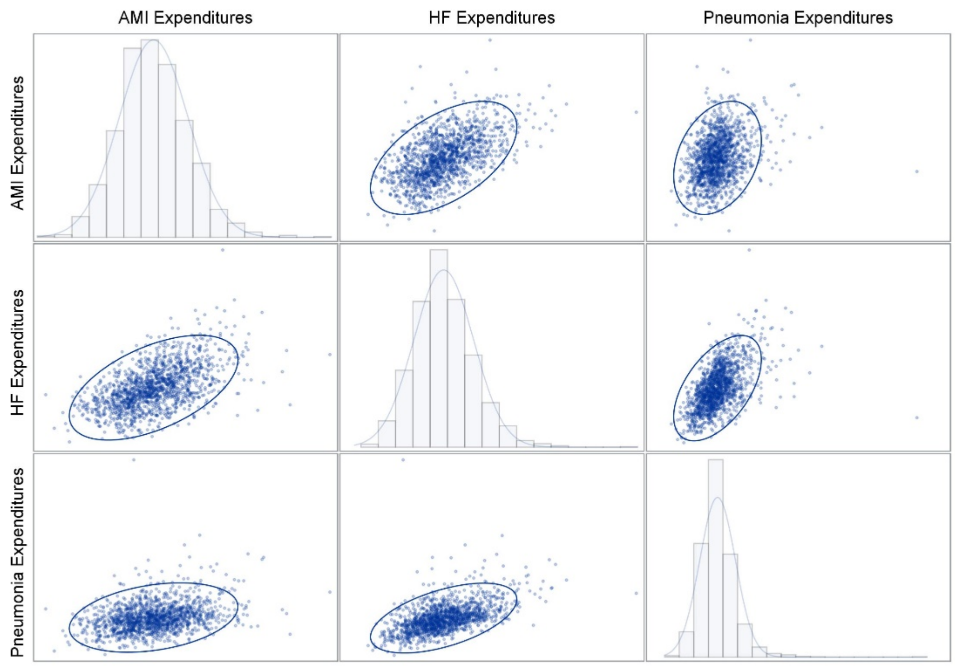

Pearson correlation coefficients: 0.57 (AMI vs. HF), 0.26 (AMI vs. pneumonia), and 0.50 (HF vs. pneumonia)
AMI=Acute Myocardial Infarction, HF=Heart Failure

© 2020 Wang Y et al. *JAMA Network Open*.

eFigure 3. Relationship of risk-standardized rate of adverse events among AMI, HF, and pneumonia

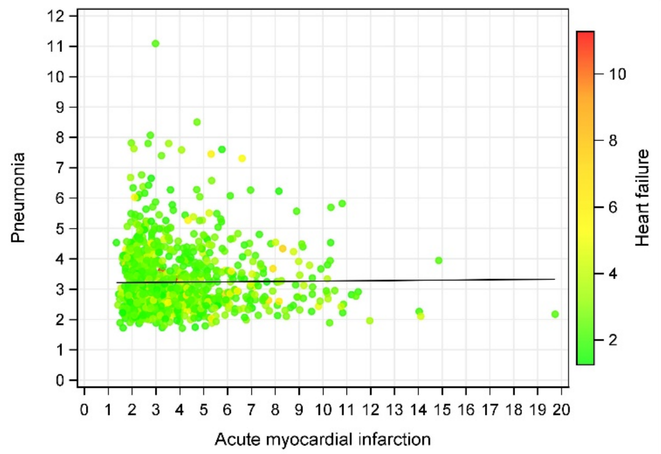

Relationship of risk-standardized occurrence rate of adverse events among three conditions
Each dot represents an individual hospital

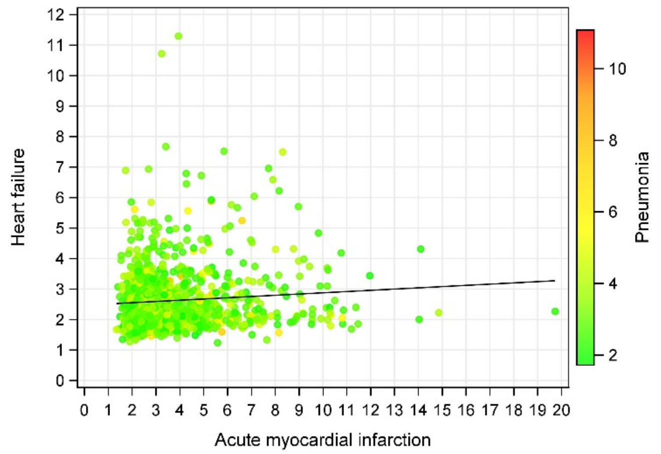

Relationship of risk-standardized occurrence rate of adverse events among three conditions
Each dot represents an individual hospital

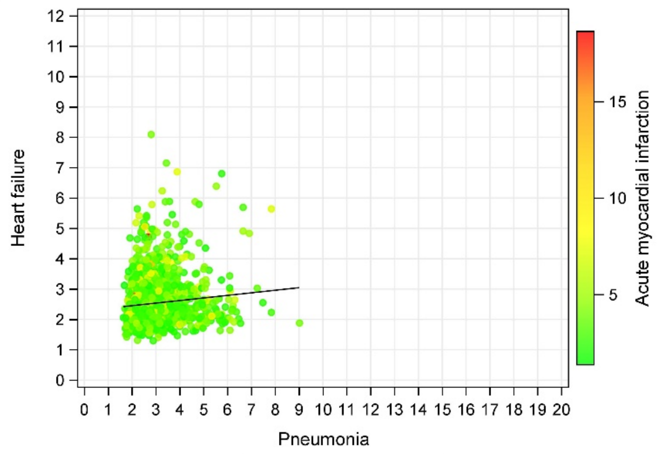

Relationship of risk-standardized occurrence rate of adverse events among three conditions
Each dot represents an individual hospital

© 2020 Wang Y et al. *JAMA Network Open*.

eFigure 4. Relationship between hospital-specific risk-standardized Medicare 30-day episode-of-care expenditures and adverse events by condition

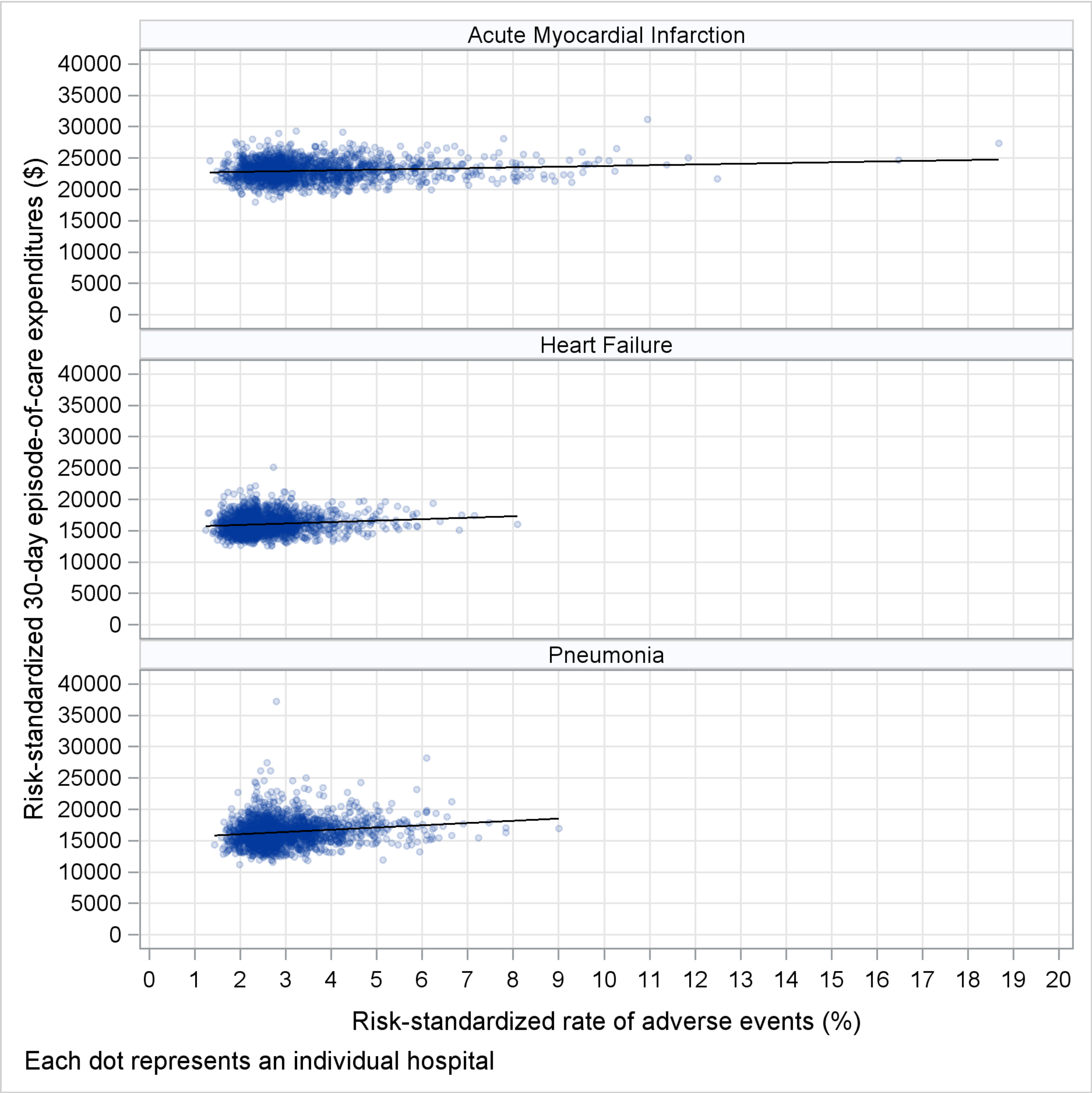

© 2020 Wang Y et al. *JAMA Network Open*.

eFigure 5. Association between hospital-specific performance on patient safety and hospital-specific performance on 30-day episode-of-care expenditures by AMI, HF, and pneumonia (hospitals with at least 25 adverse events for which patients were at risk for each condition)

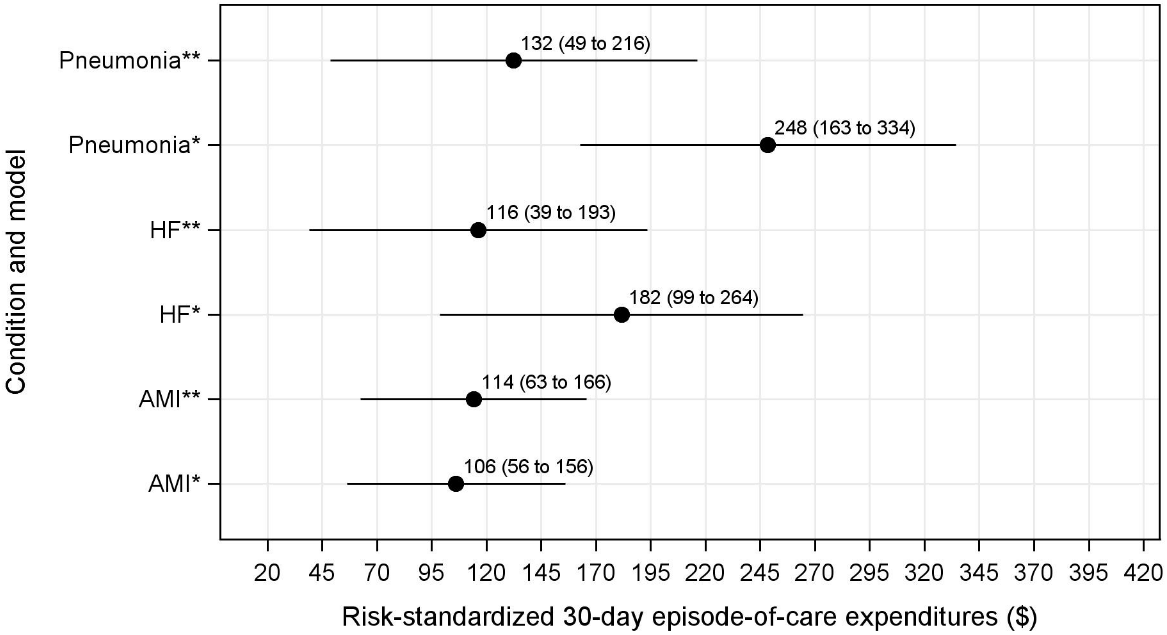

Change in 30-day episode-of-care expenditures per discharge for one percentage-point increase in adverse event rate (%)
* Unadjusted for hospital characteristics; ** Adjusted for hospital characteristics
AMI=Acute Myocardial Infarction, HF=Heart Failure

© 2020 Wang Y et al. *JAMA Network Open*.

eFigure 6. Hospital characteristics associated with high-value care

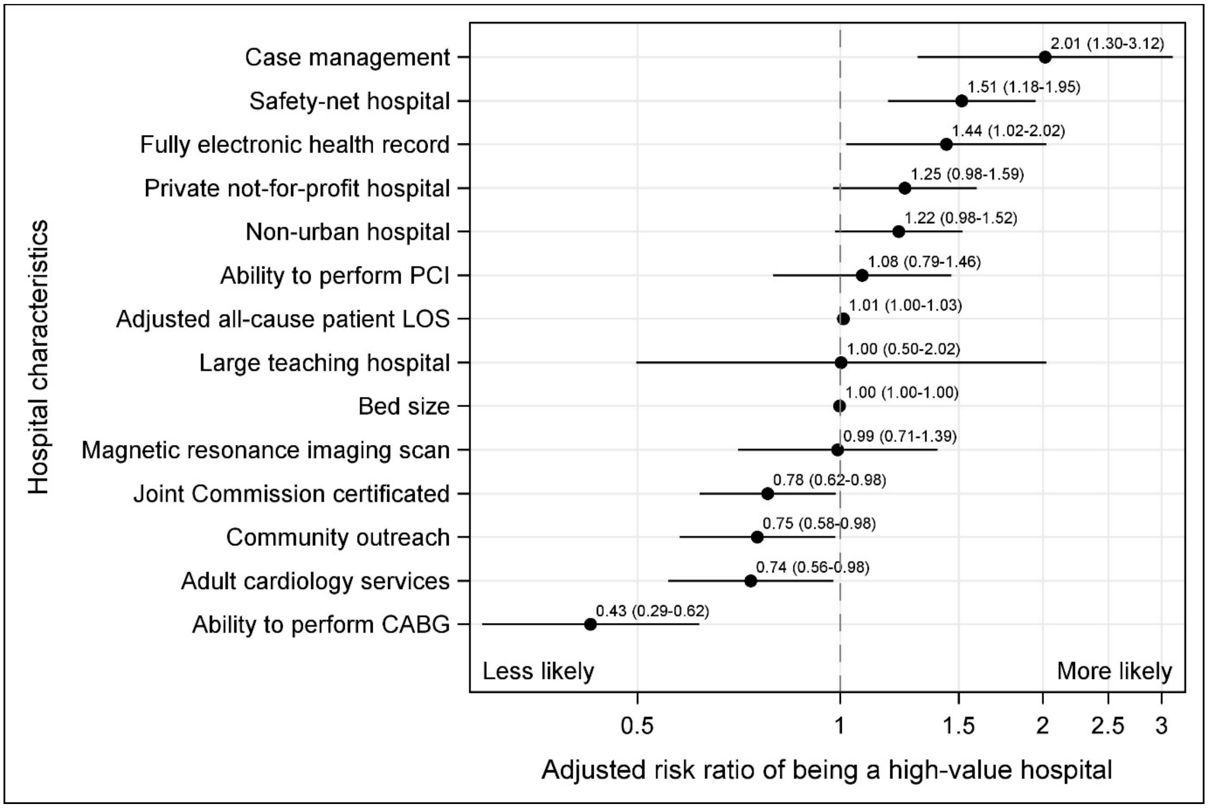

© 2020 Wang Y et al. *JAMA Network Open*.
